# Data-Driven Exploration of Selectivity and Off-Target Activities of Designated Chemical Probes

**DOI:** 10.3390/molecules23102434

**Published:** 2018-09-23

**Authors:** Filip Miljković, Jürgen Bajorath

**Affiliations:** Department of Life Science Informatics, B-IT, LIMES Program Unit Chemical Biology and Medicinal Chemistry, Rheinische Friedrich-Wilhelms-Universität, Endenicher Allee 19c, D-53115 Bonn, Germany; miljkovi@bit.uni-bonn.de

**Keywords:** chemical biology, bioactive compounds, chemical probes, target selectivity, promiscuity, molecular scaffold, off-target activity

## Abstract

Chemical probes are of central relevance for chemical biology. To unambiguously explore the role of target proteins in triggering or mediating biological functions, small molecules used as probes should ideally be target-specific; at least, they should have sufficiently high selectivity for a primary target. We present a thorough analysis of currently available activity data for designated chemical probes to address several key questions: How well defined are chemical probes? What is their level of selectivity? Is there evidence for additional activities? Are some probes “better” than others? Therefore, highly curated chemical probes were collected and their selectivity was analyzed on the basis of publicly available compound activity data. Different selectivity patterns were observed, which distinguished designated high-quality probes.

## 1. Introduction

In 2003, the Human Genome Project was completed [1,2], which catalyzed biomedical research in an unprecedented manner. Among others, the emerging field of chemical biology was spurred on through the availability of annotated gene sequences, the development of new screening techniques, and advances in computational biology [3,4]. Many newly sequenced genes and their products became available for further exploration, which also opened the door for the identification of new targets for drug discovery [5,6,7]. Studying the function(s) of candidate targets in physiological environments and under pathological conditions became a primary objective for chemical biology. For this purpose, small molecules were used as chemical probes to specifically assess consequences of target intervention for biological functions and processes [8,9,10].

Chemical probes have stringent requirements. They must be capable of selectively binding to targets and modulating protein functions in their physiological context; ideally, probes should be target-specific. Furthermore, if they are used in a phenotypic context, it must ultimately be possible to deconvolute and identify targets that are responsible for an interesting biological readout [8,11,12,13,14]. These are challenging tasks. Not surprisingly, the scientific community is continuously revising and updating requirements for high-quality probes. One example is provided by a proposal of the Structural Genomics Consortium [15], according to which a high-quality probe should have higher than 100 nM potency against its designated target and exhibit greater than 30-fold selectivity for its primary target over other proteins belonging to the same family. Moreover, for phenotypic applications, a probe should display significant cellular on-target activity at 1 μM concentration. However, more often than not, such requirements are not met by candidate compounds considered as probes [13].

In addition to defining key requirements for chemical probes, the scientific community has set out to evaluate and rate probes and validate their use in cellular or in vivo model systems. For example, this is attempted through submission of candidate compounds to the Chemical Probes Portal [13,14], which recommends probes on the basis of ratings provided by their Scientific Advisory Board (SAB), consisting of experts in medicinal chemistry, chemical biology, or pharmacology. Guided by such ratings, an external investigator should be capable of choosing the best available probe for a target of interest and acquiring it through listed vendors [14].

Despite ongoing efforts to set high standards for chemical probes, the scientific community is still facing problems due to poor characterization of small molecule modulators, improper use of probes, and outdated recommendations [8,12,13]. The characterization and dissemination of probes will likely benefit from further support. For example, from a scientific viewpoint, a data-driven assessment of chemical probes is expected to complement expert views and experimental case studies, especially since compound activity data currently grow in an unprecedented manner.

In this study, we report a comprehensive analysis of highly curated chemical probes from the Chemical Probes Portal on the basis of compound activity data available in ChEMBL [16], the major public repository of data from medicinal chemistry. Promiscuity of probes was calculated at different data confidence levels and potency thresholds. Compound selectivity was investigated and compared to reports of the Chemical Probes Portal. Applying a scaffold concept [17], activities of chemical probes and structurally analogous bioactive compounds were compared and potential off-target activities of probes were further explored via network analysis [18]. Our findings are reported in the following.

## 2. Results and Discussion

### 2.1. Qualifying Chemical Probes

Table 1 shows target classes of chemical probes as reported by the Chemical Probes Portal. A total of 67 probes are listed, for which high-confidence activity data were available in ChEMBL and at least one activity annotation for a human target with a potency of ≤10 μM. Data confidence criteria and potency thresholds applied in our analysis are detailed in the Materials and Methods section. The 67 probes were assigned to six target classes. Almost half of these probes (33) were directed against protein kinases, followed by epigenetic probes (16).

Based on the classification in Table 1, the compounds were broadly divided into probes for kinases (34) and non-kinase targets (33). This was done because kinase inhibitors are of particular interest in chemical biology (as well as drug discovery), given the key role kinases play in many signaling pathways.

Protein kinases share an adenosine triphosphate (ATP) (cofactor)-binding site that is highly conserved across the human kinome [19]. The majority of currently available kinase inhibitors are type I inhibitors directed against the conserved ATP site, making target promiscuity among such inhibitors likely [20,21,22]. However, the presence of promiscuity cannot be assumed a priori because many type I inhibitors also display apparent selectivity for a given kinase over others [20,22]. Hence, for characterizing kinase inhibitors used as chemical probes, exploring the interplay between assumed selectivity and potential promiscuity is of particular interest.

### 2.2. Selectivity Trends of Chemical Probes

For each chemical probe, the promiscuity degree (PD) was defined as the number of its unique targets on the basis of ChEMBL activity records or target annotations of the Chemical Probes Portal. Activity of a compound against multiple targets including unrelated targets is generally rationalized as promiscuity, whereas specificity implies exclusive activity against a single target. Furthermore, selectivity is best understood as activity against very few related targets, for example, a primary target and one or two others from the same family. Hence, formally it is difficult to draw a line between low levels of compound promiscuity and selectivity. However, since promiscuity also applies to increasingly large numbers of targets, it is advantageous to introduce the promiscuity degree as a measure of multi-target activity, rather than selectivity degree.

**Figure 1:**
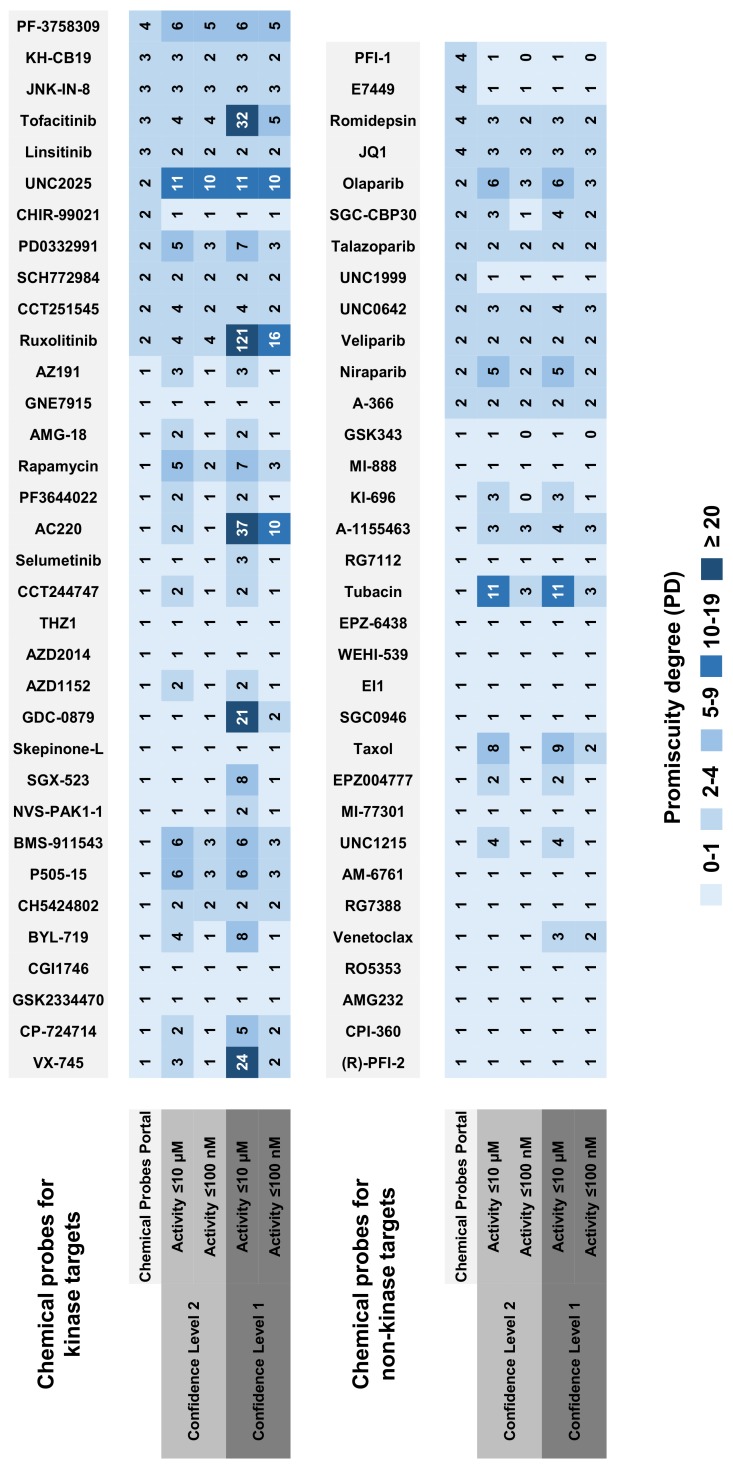
Promiscuity degree (PD) values of chemical probes are calculated on the basis of activity records from ChEMBL at two data confidence levels and for two potency thresholds and on the basis of target information from the Chemical Probes Portal. Probes for kinase and non-kinase targets are distinguished. Matrix cells represent PD values and are color-coded using a continuous spectrum ranging from light blue (0–1) to dark blue (≥20). “0” means that no target annotation is available for a given combination.

PDs were calculated on the basis of medium-confidence activity data (level 1; see Materials and Methods) and high-confidence data (level 2) applying two different potency thresholds (≤10,000 nM and ≤100 nM; see Materials and Methods). In the following, the ≤10,000 nM threshold is referred to as ≤10μM. For each probe, four PDs were obtained by combining data confidence level 1 and 2 with the two potency thresholds, and a fifth value was calculated on the basis of target annotations provided by the Chemical Probes Portal. The results obtained for the 67 probes are reported in Figure 1.

Based on the information provided by the Chemical Probes Portal, probes were active against one to four targets, with on average 1.6 targets per probe. The majority of probes only had a single target annotation, consistent with proposed high-quality probe characteristics. On the basis of activity data from ChEMBL, a somewhat different picture emerged. For the data confidence level 2/≤100 nM threshold combination, most probes retained their Portal-based PD value, yielding a similar average of 1.7 targets per probe. For the confidence level 2/≤10 μM threshold combination, nearly half of the probes retained their PD. However, for others, an increase in promiscuity was detected, yielding an average of 2.6 targets per probe. In some cases, a PD > 5 was observed. These findings indicated that a subset of probes were at least weakly active against multiple targets.

Next, data confidence criteria were relaxed and PD values calculated at confidence level 1 applying a potency threshold ≤10 μM. In this case, about half of the qualifying probes also retained their Portal-based PD value. However, for other probes a substantial increase in promiscuity was observed, resulting in an average PD of 6.3 targets per probe. When applying the more rigorous ≤100 nM threshold at level 1, the mean PD decreased again to 2.2 targets per probe, revealing that reported weak activities at medium data confidence were largely responsible for the significant increase in the average PD.

**Figure 2:**
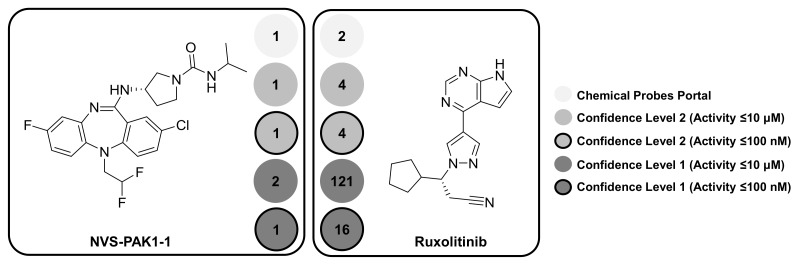
Shown are two exemplary kinase probes. For each probe, five PD values are reported applying different data selection criteria.

Taken together, the results in Figure 1 show that proposed target selectivity of about 50% of the designated high-quality probes was not altered by taking medicinal chemistry data at varying confidence levels and potency thresholds into account. This was an encouraging finding, which also applied to kinase probes having an intrinsic likelihood of multi-kinase activity. By contrast, a significant increase in promiscuity was observed for another subset of probes. Table 2 reports 10 kinase probes that were annotated with both kinase and non-kinase targets when applying the confidence level 1/≤10 μM threshold combination. Two exemplary kinase probes with a different degree of selectivity are shown in Figure 2, NVS-PAK1-1 [23] and ruxolitinib [24].

NVS-PAK1-1 [23] is an allosteric inhibitor of serine/threonine-protein kinase PAK1, as reported by the Chemical Probes Portal. Importantly, allosteric kinase inhibitors bind to regions outside the conserved ATP site and are thus expected to be more target-selective than type I inhibitors. At data confidence level 2, PAK1 was the only target of NVS-PAK1-1, regardless of the potency threshold. For the confidence level 1/≤10 μM threshold combination, only the closely related serine/threonine-protein kinase PAK2 was detected as an additional target. However, this was not the case when the ≤100 nM threshold was applied. Thus, on the basis of activity data analysis, NVS-PAK1-1 was a highly selective chemical probe, consistent with its allosteric mode-of-action and the Portal assessment.

Ruxolitinib [24] is an ATP-competitive pan-JAK inhibitor with JAK1 and JAK2 as designated primary targets. In this case, a different picture emerged. At confidence level 2 and both potency thresholds, activity of ruxolitinib was reported against two other members of the Janus kinase family, JAK3 and TYK2. Moreover, at confidence level 1, drastic increases in promiscuity were detected. At the ≤100 nM potency threshold, 16 kinase annotations were obtained and at the ≤10 μM threshold, a total of 121 targets were detected. These findings have two implications. First, promiscuity must be strictly considered in light of data confidence and potency criteria. For example, comparing the PD values obtained at confidence level 1 and 2, it is unlikely that ruxolitinib would be weakly active against more than 100 targets, and thus some of these annotations might well be false positive. Second, there was a clear difference in selectivity between NVS-PAK1-1 and ruxolitinib. Not unexpectedly, given its classification as an ATP site-directed pan-JAK inhibitor, ruxolitinib exhibited target promiscuity, and this well beyond the Janus kinase family, as revealed by our activity data analysis. Hence, the use of ruxolitinib as a pan-JAK probe might be called into question, even if a number of target annotations detected at medium data confidence and, especially, low potency are false positive. 

### 2.3. Chemical Probes and Historic Compounds

Chemical Probes Portal also reports a class of small molecules termed “historic compounds” [14]. As the name implies, many of these compounds were previously used as chemical probes, but considered to be obsolete or inferior to others at some point. Typically, historic compounds were found to be non-selective or not sufficiently potent to meet high-quality probe standards. For each historic compound, the Portal provides a rationale as to why it should not be further considered as a probe [14]. We reasoned that these historic compounds might present an interesting case for comparison with current probes.

**Figure 3:**
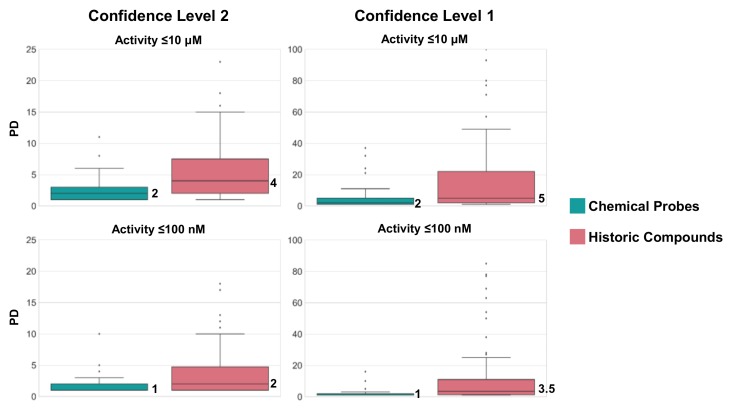
Boxplots report the distribution of PD values of qualifying chemical probes (green) and historic compounds (red) on the basis of ChEMBL data (top: Level 2 and 1, threshold ≤10 μM, chemical probes: 67, historic compounds: 127; bottom: Level 2 and 1, threshold ≤100 nM, chemical probes: 64; historic compounds: 94). Boxplots contain the smallest value (bottom line), first quartile (lower boundary of the box), median value (thick line), third quartile (upper boundary), largest value (top line), and outliers (points below the bottom or above the top line).

For the confidence level 2 and 1/≤10 μM threshold combinations, activity annotations for 127 of the 164 historic compounds of Chemical Probes Portal were identified in ChEMBL, applying the same criteria as for chemical probes. For the level 2 and 1/≤100 nM threshold combinations, activity annotations were detected for 94 historic compounds. Figure 3 compares the distribution of PD values for chemical probes and historic compounds for all four combinations.

In general, historic compounds had higher PD values than current chemical probes on the basis of available activity data, consistent with the Portal assessment. Hence, shortcomings of historic compounds were attributable to limited target selectivity.

### 2.4. Scaffold Analysis of Chemical Probes

Applying the confidence level 2/≤10 μM threshold combination, 67 chemical probes, combined with qualifying 233,675 bioactive compounds from ChEMBL and Bemis-Murcko (BM) scaffolds [17] (see Materials and Methods), were extracted from this compound set. BM scaffolds represent molecular core structures and compounds sharing the same scaffold from a series of analogs. The 67 chemical probes yielded 66 unique BM scaffolds. For each probe, bioactive compounds sharing the same scaffolds were collected and their target annotations recorded. Target annotations of structural analogs assigned to probe scaffolds provide additional target hypotheses for probes (i.e., hints at off-target activities).

**Figure 4:**
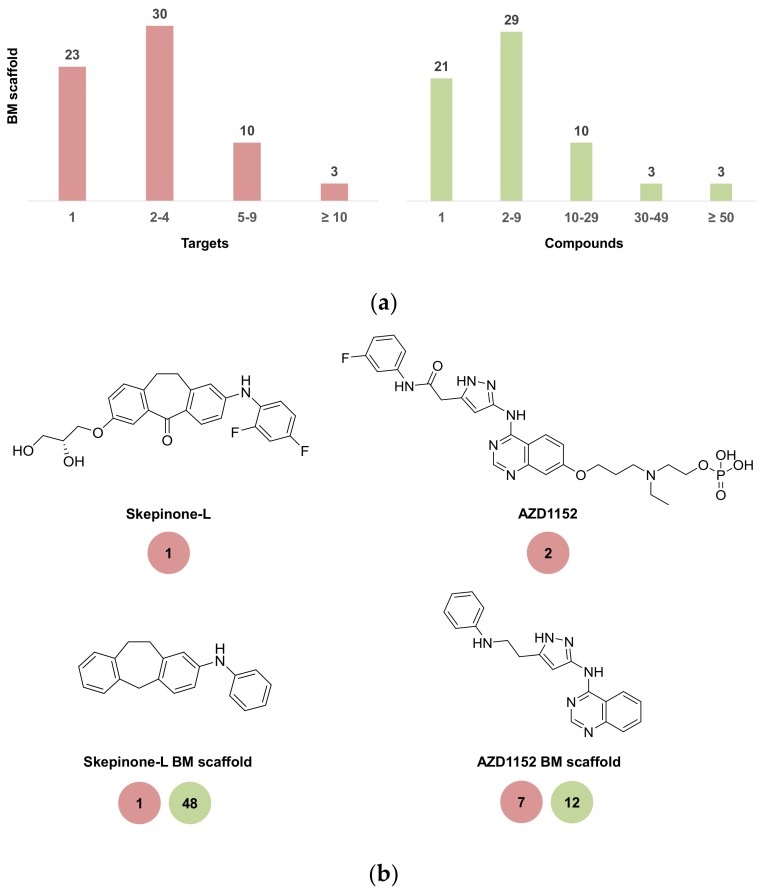

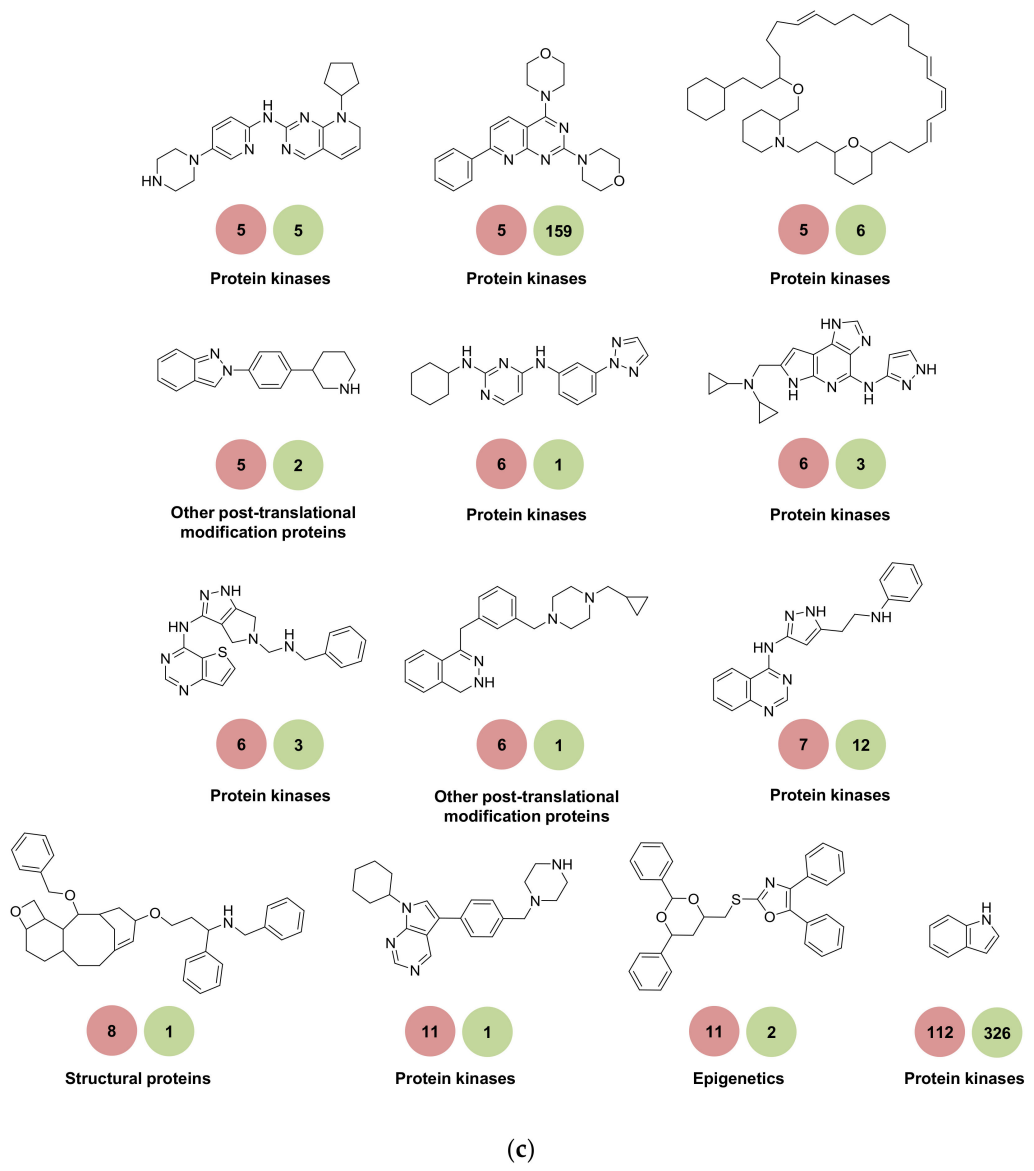
(**a**) Bar graphs report the distributions of compounds and targets over probe scaffolds. (**b**) Kinase probes skepinone-L and AZD1152 are shown together with their BM scaffolds. For these probes and their scaffolds, promiscuity degrees are compared (red background). In addition, the total number of analogs represented by each probe scaffold is given (green). (**c**) Shown are 13 chemical probe scaffolds with highest “meta-level” promiscuity (i.e., associated with five or more targets). For these scaffolds, the promiscuity degrees (red background) and number of analogs (green) representing them are reported. In addition, classes of primary targets of compounds containing the scaffolds are given as reported by Chemical Probes Portal.

Figure 4a shows the distribution of target annotations (red) and ChEMBL compounds (green) over the 66 BM scaffolds extracted from probes. Only small numbers of bioactive compounds contained probe scaffolds. For 21 scaffolds, no additional ChEMBL compounds were identified (i.e., these scaffolds exclusively represented the probe). For 29 other scaffolds, a total of two to nine analogs (including the probe) were identified. Only six probe scaffolds represented 30 or more analogs. Thus, chemical probes frequently contained unique core structures. Since only limited numbers of analogs were detected for the majority of probes, the number of cumulative target annotations per probe scaffold was overall also small. The majority of scaffolds (53 of 66) were associated with one to four targets and only three scaffolds with 10 or more targets. Thus, “meta-level” promiscuity of chemical probe scaffolds was also low.

Figure 4b shows two kinase chemical probes and their BM scaffolds. Skepinone-L [25,26] is an ATP-competitive MAP kinase p38 alpha inhibitor with an unusual binding mode. Its scaffold represented a total of 48 analogs, all of which were exclusively annotated with MAP kinase p38 alpha. Hence, skepinone-L is another highly selective kinase probe. AZD1152 [27,28] is a phosphate-containing pro-drug that is rapidly converted in vivo into an active alcohol. Chemical Probes Portal reports the active form of AZD1152 as a selective inhibitor of serine/threonine kinase Aurora-B. In ChEMBL, an additional target was found for AZD1152. Moreover, although the scaffold of AZD1152 only represented 12 analogs—much less than the skepinone-L scaffold—these analogs were active against a total of seven targets, indicating that AZD1152 was likely less selective than proposed. Thus, skepinone-L and AZD1152 represent another example of designated high-quality probes with notable differences in selectivity revealed by activity data analysis, similar to NVS-PAK1-1 and ruxolitinib shown in Figure 2. The set of 13 structurally diverse scaffolds with highest “meta-level” promiscuity is shown in Figure 4c. Compounds containing these scaffolds were active against a total of five or more targets.

### 2.5. Off-Target Activity Assessment in Networks

Going beyond scaffold analysis, similarity relationships between chemical probes and other bioactive compounds can also be explored on the basis of matched molecular pair (MMP) analysis [29]. An MMP is defined as a pair of compounds that are only distinguished by a chemical change at a single site. MMPs can be efficiently generated algorithmically. As another criterion of structural similarity [30], a chemical probe and bioactive compounds were classified as similar if they formed an MMP. Such structural relationships can be conveniently displayed in molecular networks in which nodes represent compounds and edges account for pairwise MMPs. If one assigns different node types to chemical probes and other bioactive compounds, a bipartite network is obtained, which can be further extended to a tripartite design by adding targets as a third node category. In the resulting tripartite network, edges between probes and analogs represent similarity relationships and edges between compounds and targets activity relationships. For chemical probes with bioactive analogs (connected by edges) additional targets associated with these analogs can be considered since it can be assumed that probes are likely to also be active against targets of structurally closely related compounds. 

**Figure 5:**
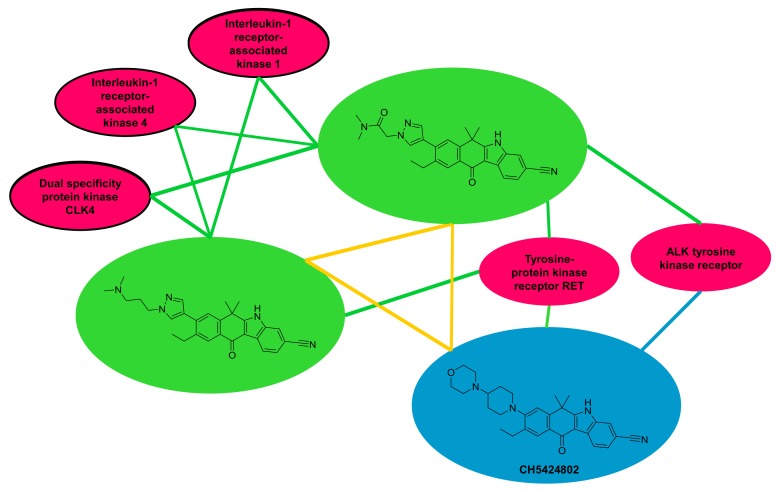
Shown is the neighborhood of a chemical probe (CH5424802) in a tripartite network. Blue and green nodes represent probes and bioactive analogs, respectively, and red nodes targets. Blue edges represent interactions between probes and targets from the Chemical Probes Portal, and green edges interaction between all compounds and targets from ChEMBL. In addition, yellow edges indicate MMP (similarity) relationships. Targets of analogs that provide additional hypotheses for the probe are encircled. This network should be considered “pseudo-tripartite” because the formation of edges (MMP relationships) is also permitted here between nodes belonging to the same category, departing from fundamental network theory. The network was drawn with Cytoscape 3.6.1. using the “organic layout” function [32].

MMPs were obtained for 49 of 67 chemical probes and 738 bioactive analogs from ChEMBL. These compounds were active against a total of 135 targets. A tripartite network was constructed to capture all structural and activity relationships. The network revealed 47 previously unobserved probe-target associations involving a subset of 16 chemical probes and 40 targets from ChEMBL. New relationships can be studied in a structure-activity context by focusing on network neighborhoods of chemical probes. An example is provided in Figure 5, which shows the network neighborhood of CH5424802 [31], an ATP-competitive inhibitor of ALK tyrosine kinase, as reported by the Chemical Probes Portal, for which an additional target, RET tyrosine kinase, was identified in ChEMBL. CH5424802 had two close structural analogs with reported activity against the same and other kinases, thus providing additional target hypotheses for the chemical probe.

### 2.6. Summary

Small molecular probes are of central importance to chemical biology. However, many currently investigated probes remain to be fully characterized. To these ends, important contributions are made by the Chemical Probes Portal, which carefully assesses candidate probes and prioritizes a set of highly curated chemical probes. Herein, we have further investigated designated high-quality probes by systematic analysis of available activity data for probes and closely related bioactive compounds. Our analysis adds another layer to the characterization of probes for chemical biology. Taking different data confidence and potency criteria into account, we show that ~50% of designated high-quality probes are target-selective when all available activity data are considered, consistent with expert curation; an encouraging finding. This applies to chemical probes directed against kinase or non-kinase targets. On the other hand, activity data analysis also differentiates between probes and identifies a subset of putative high-quality probes for which selectivity cannot be supported on the basis of currently available data, as summarized in Figure 1. These chemical entities might be deprioritized and should be used with caution when exploring biological functions and their origins. However, the analysis also emphasizes the presence of a variety of probes with striking selectivity—including kinase inhibitors—indicating that further progress in generating high-quality chemical probes can be anticipated, which will be exciting to follow.

## 3. Materials and Methods

### 3.1. Chemical Probes

Chemical probes were extracted from Chemical Probes Portal (accessed in July 2018) [13,14], which reports 189 small molecule modulators for applications in biomedical research. From this collection, only probes were selected that (i) were classified as inhibitors of a primary target, (ii) had non-ambiguous SMILES representations, (iii) were associated with ChEMBL identifiers (ChEMBL IDs) [16], and (iv) had a sufficiently high rating. The last selection criterion requires further explanation. Members of the Chemical Probes Portal SAB assign priority star ratings (1–4 stars) to probe candidates for their application in cellular and/or in vivo models. A star rating of 1 indicates that a candidate cannot be recommended as a probe, whereas a rating of 4 represents a high recommendation. Expert ratings for a candidate compound are averaged to obtain a final consensus rating. The Chemical Probes Portal endorses compounds as chemical probes only if their final rating reaches at least 3 stars [14]. Therefore, only candidate probes with a final rating of at least 3 stars were selected for our analysis. On the basis of selection criteria (i)–(iv), 80 highly curated probes qualified for our analysis.

### 3.2. Activity Data, Confidence Levels, and Historic Compounds

For selected chemical probes, available activity data for human targets were extracted from ChEMBL (release 24). Activity data were evaluated at two different confidence levels including level 1 (medium confidence) and level 2 (high confidence) according to [21]. For level 1, activity data with highest assay confidence were required, i.e., activity annotations were only selected from direct inhibition assays (ChEMBL assay relationship type “D”) for single targets at the highest assay confidence level (“9”). For level 2, activity data with highest assay confidence plus highest measurement confidence were selected. Highest measurement confidence required the availability of numerically specified standard activity measurements (K_i_ or IC_50_ values with “=” standard relation), use of the nanomolar (“nM”) activity unit, and presence of fully consistent “activity comments” in ChEMBL. To investigate selectivity characteristics of chemical probes, two potency thresholds were applied to activity data at confidence levels 1 and 2, i.e., ≤10,000 nM (≤10μM) and ≤100 nM. For each chemical probe, PD values were calculated for each combination of a confidence level and potency threshold, yielding four PD values per probe from ChEMBL data. An additional PD value was calculated on the basis of target annotations reported by the Chemical Probes Portal. At confidence level 2 applying the ≤10 μM potency threshold, at least one activity record for a human target was obtained for 67 of 80 pre-selected chemical probes. These 67 probes provided our basis set for subsequent analysis. We also searched ChEMBL for 164 historic compounds designated by the Chemical Probes Portal on the basis of the same criteria applied to chemical probes.

### 3.3. Bioactive Compounds, Scaffold Analysis, and Off-Target Predictions

Scaffold analysis of chemical probes was performed applying the Bemis-Murcko (BM) scaffold concept [17]. BM scaffolds are extracted from compounds by eliminating all R-groups while retaining ring systems and linker moieties connecting rings. So-defined scaffolds were derived from all chemical probes and bioactive compounds for which target annotation(s) were available at confidence level 2 applying the ≤10 μM potency threshold. Potential off-target activities of chemical probes were also analyzed using a tripartite network data structure [18].

## Figures and Tables

**Table 1:** The table reports designated target classes of chemical probes from the Chemical Probes Portal, for which qualifying activity data were available in ChEMBL.

Target Class	Chemical Probes	Target-Based Categories
Protein kinases	33	Chemical probes for kinase targets
Lipid kinases	1	
Epigenetics	16	Chemical probes for non-kinase targets
Other post-translation modification proteins	13	
Other proteins	3	
Structural proteins	1	

**Table 2:** Reported are kinase probes that are annotated in ChEMBL, with both kinase and non-kinase targets.

Chemical Probe	Confidence Level 2		Confidence Level 1	
	≤10 μM	≤100 nM	≤10 μM	≤100 nM
BMS-911543	6 (2) ^1^	3	6 (2)	3
BYL-719	4 (2)	1	8 (3)	1
CCT244747	2 (1)	1	2 (1)	1
CCT251545	4 (1)	2 (1)	4 (1)	2 (1)
P505-15	6 (1)	3	6 (1)	3
PF3644022	2 (1)	1	2 (1)	1
Rapamycin	5 (4)	2 (1)	7 (6)	3 (2)
Ruxolitinib	4	4	121 (1)	16
SGX-523	1	1	8 (1)	1
VX-745	3 (2)	1	24 (2)	2

^1^ Probes were selected if non-kinase targets were available for at least one confidence level/threshold combination. For each combination, promiscuity degree (PD) values are reported. If non-kinase targets are available, their number is given in parentheses. For example, “2 (1)” means that the probe is annotated with two targets, including one kinase target and “2” that the probe is annotated with two kinases (and no non-kinase target).
